# Resveratrol Delivery from Implanted Cyclodextrin Polymers Provides Sustained Antioxidant Effect on Implanted Neural Probes

**DOI:** 10.3390/ijms21103579

**Published:** 2020-05-19

**Authors:** Rebecca M. Haley, Sean T. Zuckerman, Hassan Dakhlallah, Jeffery R. Capadona, Horst A. von Recum, Evon S. Ereifej

**Affiliations:** 1 Department of Biomedical Engineering, Case Western Reserve University, Cleveland, OH 44106, USA; rmh118@case.edu (R.M.H.); jrc35@case.edu (J.R.C.); 2 Affinity Therapeutics, LLC, 11000 Cedar Avenue, Suite 285, Cleveland, OH 44106, USA; stzuckerman@gmail.com; 3 Veteran Affairs Ann Arbor Healthcare System, Ann Arbor, MI 48105, USA; hassdak@umich.edu; 4 Advanced Platform Technology Center, Louis Stokes Cleveland Veterans Affairs Medical Center, Cleveland, OH 44106, USA; 5 Department of Biomedical Engineering, University of Michigan, Ann Arbor, MI 48109, USA; 6 Department of Neurology, University of Michigan, Ann Arbor, MI 48109, USA

**Keywords:** oxidative stress, intracortical electrode, resveratrol, drug delivery, cyclodextrin

## Abstract

Intracortical microelectrodes are valuable tools used to study and treat neurological diseases. Due in large part to the oxidative stress and inflammatory response occurring after electrode implantation, the signal quality of these electrodes decreases over time. To alleviate this response, resveratrol, a natural antioxidant which elicits neuroprotective effects through reduction of oxidative stress, was utilized. This work compares traditional systemic delivery of resveratrol to the novel cyclodextrin polymer (pCD) local delivery approach presented herein, both in vitro and in vivo. The pCD displayed an extended resveratrol release for 100 days, as well as 60 days of free radical scavenging activity in vitro. In vivo results indicated that our pCD delivery system successfully delivered resveratrol to the brain with a sustained release for the entire short-duration study (up to 7 days). Interestingly, significantly greater concentrations of resveratrol metabolites were found at the intracortical probe implantation site compared to the systemic administration of resveratrol. Together, our pilot results provide support for the possibility of improving the delivery of resveratrol in an attempt to stabilize long-term neural interfacing applications.

## 1. Introduction

Intracortical microelectrodes (IME) are a neuroscience tool that can be utilized for studying the functions of the nervous system [1]. IMEs can also be used to treat neurological diseases and injuries. There has been a large spread of advancements utilizing neuronal communication through brain computer interface systems to restore limited motor functions from those with spinal cord injury and amyotrophic lateral sclerosis (ALS) [2]. Unfortunately, the signal quality from electrophysiology recordings has been shown to decrease over time, in part due to the inflammatory and oxidative stress responses around the electrode. During implantation, breaching of the blood–brain barrier occurs, resulting in the infiltration of neurotoxic factors and myeloid cells contributing to the inflammatory response [3]. Microglia and macrophage activation follows, exhibiting a frustrated phagocytic response towards the implanted electrode, and releasing proinflammatory cytokines [4,5], ensuing in reactive astrocytes migration to the implantation site and encapsulation of the device by forming the “glial scar” [4]. Concurrently, oxidative stress demonstrated by the release of reactive oxygen species (ROS) and reactive nitrogen species (RNS) by glial cells, has also been identified as a factor contributing to the failure of IMEs [6,7,8,9]. Oxidative stress leads to neuronal death, exacerbation of the inflammatory response, and corrosion of the electrode [8,9,10,11]. There is a considerable impact from ROS and RNS to the brain, due to the biochemical composition containing an abundance of unsaturated lipids [12]. Unsaturated lipids are targets of free radicals attacks, as well as are highly susceptible to oxidative modification and lipid peroxidation [12]. Additionally, neurons have low antioxidant activity levels and high methyl ions, resulting in a vulnerability to the damage of cellular lipids, proteins, and DNA [12,13]. 

Fortunately, the use of antioxidants can reduce oxidative stress and the incidence rate of neuronal damage [14]. Natural antioxidants, such as blueberries and berberine, have shown anti-inflammatory properties resulting in preservation of myelin in Multiple Sclerosis and inhibition of pathogenic enzymes in Alzheimer’s Disease, respectively [15,16]. Resveratrol, a polyphenol highly concentrated in red wine and grapes, has many pharmacological properties, including antioxidant, anti-inflammatory, anticancer effects, and prevention of cardiovascular disease [17,18,19,20,21,22,23,24]. Resveratrol has also been found to be a neuroprotectant against ischemia, seizure, Alzheimer’s disease, Parkinson’s disease, Huntington’s disease, and traumatic brain injury (see [25] for a good review). Resveratrol exists in two isomer forms (cis-resveratrol and trans-resveratrol), with trans-resveratrol having higher biologically active properties, thus it is commonly utilized as a therapeutic [26,27,28]. Previous work by the Capadona laboratory, investigating the use of resveratrol to mitigate oxidative stress and neuroinflammation around implanted intracortical probes, found that acute intraperitoneal administration of resveratrol had reduced ROS accumulation and blood–brain barrier instability, and increased density of neurons around the implant site at two weeks, but not at later time points [10]. Thus, the evaluation of chronic daily administration of resveratrol was investigated [29]. Daily administration of resveratrol resulted in limited levels of ROS and neurodegeneration around the electrode at two weeks post implantation, and qualitatively less neurodegeneration at 16 weeks [29]. However, abdominal adhesions and hemorrhaging around the injection site resulting from the chronic repeated intraperitoneal delivery of resveratrol led to the optimization of intraperitoneal resveratrol delivery [29].

The diluent and frequency of intraperitoneal injections was optimized to 30 mg/kg of resveratrol in either 5% ethanol in corn oil or 10:90 propylene glycol and corn oil diluent at a frequency of either biweekly IP administration or every other day, respectively. Histological findings demonstrated significantly more astrocyte reactivity at two weeks post implantation from animals receiving IP injections of resveratrol every other day compared to control animals not receiving any injections (unpublished data, Figure S1). Similar work has shown that systemic intraperitoneal delivery of single-walled carbon nanotubes can result in neuronal damage through oxidative stress and inflammation in the brain [30]. Furthermore, propylene glycol was discovered to have epileptic side effects on the rats and was discontinued from future animal studies [31]. Therefore, the frequency of IP delivery of resveratrol in a 5% ethanol in corn oil diluent was reduced to every two weeks, in which electrophysiology and bioavailability were investigated. Unfortunately, due to resveratrol’s poor solubility and blood–brain barrier permeability (unpublished data, Figure S2A), it was discovered that there were no significant differences in recording quality between control animals receiving no resveratrol and animals receiving IP injections of resveratrol every two weeks, suggesting that a more ideal mechanism of delivery be established (unpublished data, Figure S2B). Accordingly, it was concluded that improvements in histology, bioavailability and electrophysiology would not be achieved utilizing systemic delivery of resveratrol, and hence, the investigation of localized delivery methods began. 

Localized release of resveratrol would allow for a reliable method of dosing, while also limiting the complications that come along with systemic intraperitoneal administration. Previous work from the Capadona lab utilizing a compliant nanocomposite material doped with resveratrol resulted in a reduction in activated microglia/macrophages and increased neuron density around the implant site at two weeks post implantation, but no differences at more chronic time points due to the short-term release of the antioxidant [32]. Accordingly, significant time was invested in developing the cyclodextrin-based polymeric drug delivery system described herein to allow for long-term, sustained release of resveratrol. 

Soluble, free cyclodextrin (CD) is a widely used constituent in pharmaceutical and FDA-approved applications, including foods, cosmetics, and therapeutics [33,34]. CD-based materials are attractive for biomedical use due to being highly biocompatible, demonstrating low toxicity and immunogenicity in long-term release applications [35,36]. Specific to our current study, CD has been used for neuronal applications with no associated neurotoxicity [37,38,39]. In fact, CDs have been found to be a neuroprotectant and reduce neurotoxicity [37,38,39]. In the pharmaceutical industry, soluble, free CDs have been used to overcome issues of solubility and stability for a number of therapeutics, resulting in numerous FDA-approved formulations incorporating CDs [40,41]. This study exploits both CDs good biocompatibility and stable host/guest complexation to make a polymerized cyclodextrin (pCD) as a carrier of resveratrol, and evaluates its capacity for long-term release profiles both in vitro and in vivo. The findings from this current study present a proof of concept approach to locally deliver resveratrol to the neural probe implantation site utilizing a novel polymerized cyclodextrin disk loaded with resveratrol. 

## 2. Results

### 2.1. Cyclodextrin–Resveratrol Binding 

**Figure 1:**
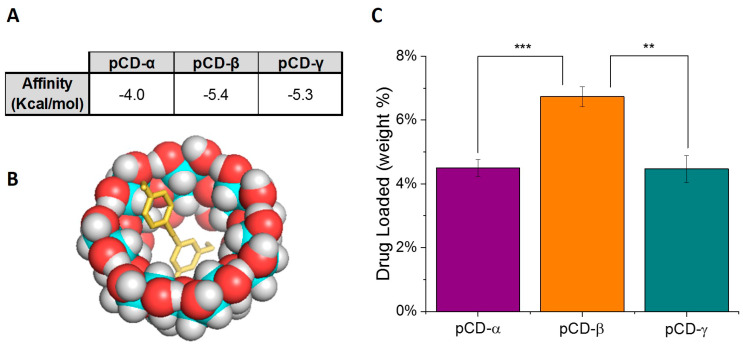
Beta-cyclodextrin demonstrates the highest affinity for resveratrol loading. (**A**) Estimated affinity binding strengths (affinity, Kcal/mol) for cyclodextrin (CD)/resveratrol complex, developed using PyRx modelling software. (**B**) 3D conformer representing the CD/resveratrol complex model. The 3D image depicts simulated resveratrol complexing with a single beta-CD molecule. (**C**) Loading ratios of resveratrol into pCD disks. Experimental studies indicated beta-CD polymer had the highest loading of resveratrol at 6.7 weight%. ** represents *p* < 0.01 and *** represents *p* < 0.001.

To establish an expected affinity release profile from CD polymers, modelling software was used to characterize the energy associated with CD/resveratrol (host/guest) inclusion complexes [42]. Using PyRx modelling software, the binding energy of resveratrol with CD was estimated with three forms of monomer CD (alpha, beta, and gamma-CD) (Figure 1A). A visual representation of this model (with beta-CD monomer as the host) can be seen in Figure 1B. Affinity was estimated to be −4, −5.4, and −5.3 Kcal/mol for alpha, beta, and gamma-CD respectively. Alpha, beta, and gamma-CD have increasing numbers of glucose residues respectively, increasing the pocket size from ~5 to ~8 angstroms, thereby slightly altering the host/guest interaction. Based on these estimates, resveratrol was found to have similar highest affinity for beta-CD and gamma-CD. 

Experimental studies confirmed that the CD/resveratrol host/guest complex allows for resveratrol loading in the polymerized form of CD as well. The maximum drug, in weight percent, that can be loaded into pCD, as well as released from pCD disks, is reported for each CD monomer type (alpha, beta, and gamma-CD) in Figure 1C. Experimental results agree with and supplement the PyRx model, indicating that pCD-beta can load the highest weight percentage of resveratrol that is available for release under in vitro drug sink conditions.

### 2.2. Polymerized Cyclodextrin–Resveratrol Release In Vitro 

**Figure 2:**
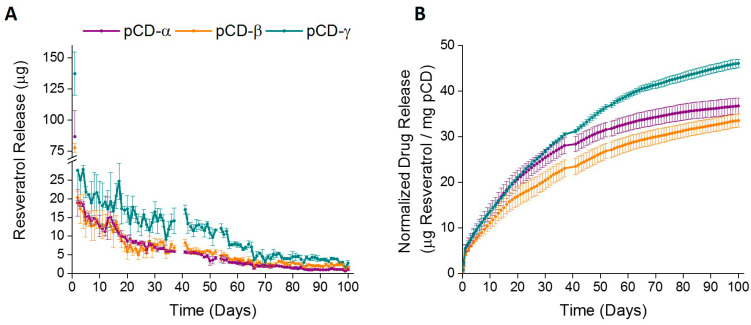
Polymerized cyclodextrin disks give long-term resveratrol release in vitro. (**A**) Alpha, Beta and Gamma pCD types displayed a small burst followed by a sustained release up to 100 days. Gamma-CD polymer exhibited the highest amount of total resveratrol released and a higher resveratrol release at all time points. (**B**) Release of resveratrol from pCD disks, normalized to CD polymer weight. Daily release of resveratrol from pCD disks displayed steadily decreasing daily dosage over 100 days of release.

Three forms of pCD (using lightly crosslinked alpha, beta, and gamma-CD prepolymers), were evaluated in vitro through drug release studies to determine the optimal form of pCD for long-term, consistent resveratrol delivery. Under physiological conditions in vitro, resveratrol was released from all pCD disk types at low but consistent dosages for 100 days after a small initial burst within the first 24 h (Figure 2). In the first 24 h, pCD disks (alpha, beta, and gamma) release 87, 78, and 137 μg (11%, 6%, and 12% of their total loaded drug) respectively (Figure 2A). After this first day, daily release slowly lowers, with an average daily rate of 17, 16, and 24 μg/day respectively in the first week and 12, 11, and 18 μg/day in the first month. By the last week of the in vitro study (days 93–100), the average daily rates dropped to 1.1, 2.1, and 3.2 μg/day respectively. Since each pCD type has varying release rates, the total amount of resveratrol released was normalized to the weight of the pCD sample (Figure 2B). Interestingly, gamma-CD polymers exhibited a higher daily release of resveratrol, as well as higher total release over time. Therefore, gamma-CD was identified as the optimal CD polymer form for long-term sustained resveratrol release utilized for in vivo studies.

### 2.3. In Vitro Antioxidant Activity of pCD Loaded with Resveratrol 

The antioxidative activity of resveratrol loaded into pCD disks was assessed using a DPPH scavenging assay. Resveratrol released from loaded pCD under physiological conditions in vitro, displayed sustained free radical scavenging activity for 50 days. A decrease in the scavenging activity began to occur after 50 days in vitro, correlating to the decrease of resveratrol concentration over time observed in Figure 2. There were no significant differences of scavenging activity between the three forms of pCD. 

**Figure 3:**
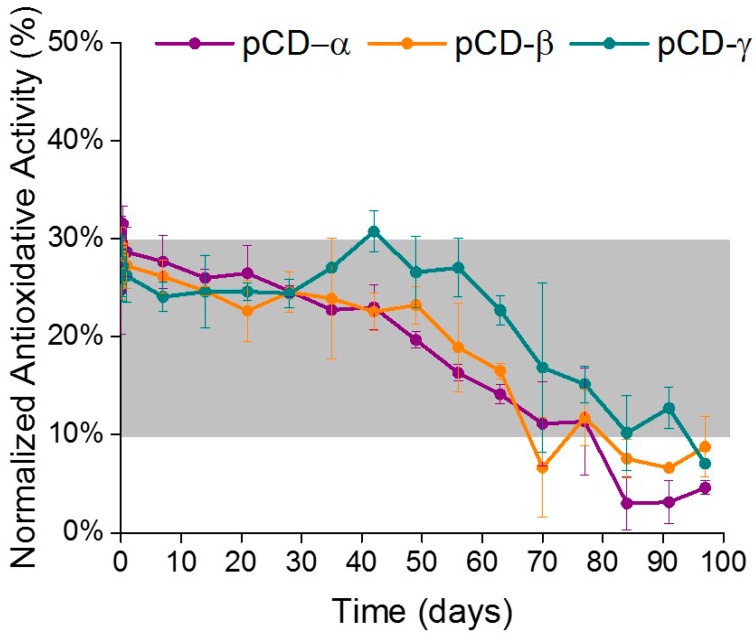
Resveratrol released from pCD shows antioxidant activity in vitro. Resveratrol displays sustained free radical scavenging activity for 50 days in vitro. The scavenging activity decreased over time, correlating to the decrease of resveratrol concentration released from pCD disks. The shaded area depicts the antioxidant activity from a standard curve using 1–100 µg/mL free resveratrol, relating to the concentrations of pCD released resveratrol.

There was an average of 10–20 μg/day of resveratrol released from the pCD during the first 30 days. The average antioxidative activity during days 1–30 was 30%, which matched the standard curve for 10 μg of free resveratrol also having 30% antioxidative activity (depicted by the shaded area in Figure 3). Similarly, there was an average of 1–3 μg/day resveratrol released during days 60–100 of the in vitro experiments, which had an average of 10% antioxidative activity. These findings also compared to the standard curve depicting 1.25 µg of resveratrol having an antioxidative activity of 12% (shaded area in Figure 3). The results demonstrate that the decrease in antioxidative activity observed around day 60 is due to the decreased concentrations of released resveratrol, rather than a decline or deficiency of activity. 

### 2.4. In Vivo Bioavailability of Resveratrol 

**Figure 4:**
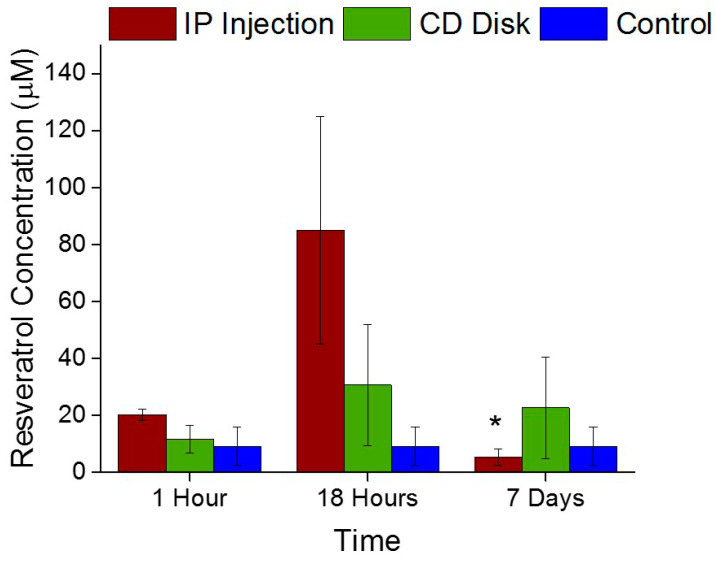
Mass Spectrometry of resveratrol. There was a significant decrease (* represents *p* = 0.05) of resveratrol concentration from 18 h to 7 days from IP administration. There was a sustained release of resveratrol from the pCD loaded disks up to 7 days in vivo.

Mass Spectrometry analysis of tissue extracted adjacent to the neural probes indicated low bioavailability of resveratrol from the IP injection and the pCD disk one hour post implantation (Figure 4). There was a non-significantly higher concentration of resveratrol around the implant from animals receiving IP administration at 18 h compared to the resveratrol loaded into pCD disks. However, there was a significant decrease (*p* = 0.05) of IP administered resveratrol concentration from 18 h to 7 days. There were no significant decreases of resveratrol concentration from pCD disks, indicating a sustained and controlled release of resveratrol from the pCD loaded disks. 

### 2.5. In Vivo Resveratrol Metabolite Bioavailability

**Figure 5:**
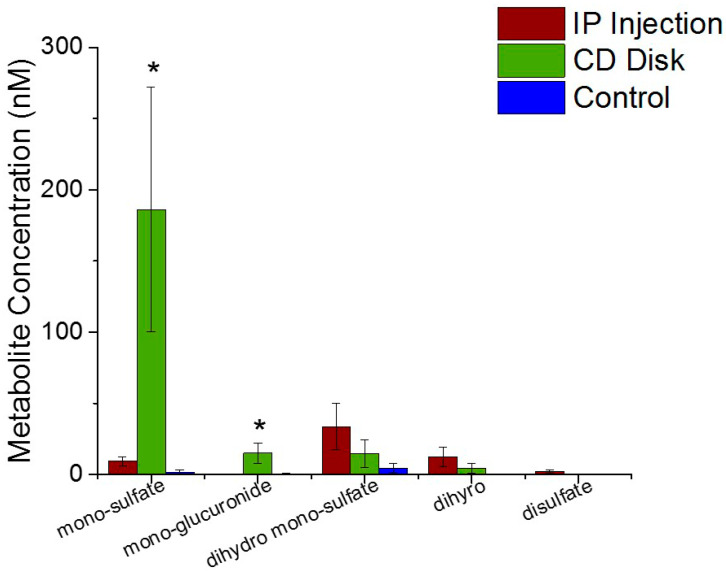
Mass Spectrometry of Resveratrol Metabolites. There was significantly more monosulfate (* represents *p* = 0.05) and mono-glucuronide (*p* = 0.05) from pCD disk compared to the IP administration.

Mass Spectrometry analysis was performed to quantitatively assess the resveratrol metabolites around the neural probes at 18 h post implantation (Figure 5). There were significantly more monosulfate (*p* = 0.05) and mono-glucuronide (*p* = 0.05) around the implant site from animals receiving resveratrol through the pCD disk compared to the IP injection. There were no significant differences of dihydro monosulfate, dihyro, and disulfate found between the treatment groups. 

## 3. Discussion

Oxidative stress has been linked as a contributor to the failure of intracortical microelectrodes [6,7,9]. Antioxidant strategies, in particular resveratrol, have been employed to mitigate the severity of damage associated with oxidative stress, but unfortunately, are hindered due to low bioavailability and ability of the antioxidant to penetrate past the blood–brain barrier [10,29]. Although the adsorption of systemic administration of resveratrol may be high, due to the extensive metabolism of resveratrol in the intestine and liver, the bioavailability of resveratrol is extremely low [43]. Intraperitoneal delivery of resveratrol may also result in an unknown amount of a drug in the circulatory system, which can cause the cerebral uptake to be slow and decreasing [30,44,45]. Moreover, systemic intraperitoneal delivery has shown negative side effects in the abdominal cavity, as well as increased inflammation and oxidative stress in the brain [29,30]. Perhaps the exacerbated inflammatory response observed in the brain is connected to the inflammation observed in the abdomen attributed to the repeated IP injections [46]. Approaches to circumvent these complications incorporated methods for local delivery of resveratrol through doping into a compliant nanocomposite material or loading into polylactic acid coated nanoparticles, which although promising, caveats included a short-term release or has yet to be investigated in vivo, respectively [32,47]. Hence, the need for a local delivery method of resveratrol, which has a long-term, sustained release. Herein, we introduced the use of polymerized cyclodextrin as a carrier for resveratrol. 

Cyclodextrins are cyclic oligosaccharides comprised of six or more glucose residues which come together to create a truncated cone shape. This cone- or ring-shaped structure allows the molecule to act as a ‘pocket’ for holding small molecule drugs. The inner space of the pocket is hydrophobic, allowing poorly soluble drug molecules, such as resveratrol, to have a high affinity for localizing in this area, thus creating a reversible host/guest inclusion complex. The insolubilization of CD can be achieved by forming a high molecular weight polymer, in which the inclusion complexes can be exploited to alter the rate of release beyond the rate of diffusion alone. The von Recum lab has shown the affinity within the inclusion complex can reduce the initial drug burst release, allowing the release of the drug to be delivered over a long period of time [36,48,49,50,51,52]. This time scale is dependent on a number of factors, but when loaded with antibiotic drugs, such as rifampicin, release from pCD disks has been recorded at antimicrobial levels for as long as 8 months [36]. The resulting release profile will be a consistent and sustained drug release, aligning with the need for long-term antioxidant delivery at the implantation site of intracortical electrodes. 

Previous work demonstrated various formulations of cyclodextrin ability to improve resveratrol biodistribution, utilizing the CD/resveratrol inclusion complex to overcome resveratrol’s low aqueous solubility and bioavailability [53,54,55]. However, the majority of these formulations incorporated the monomer form of CD (either natural or modified), which unfortunately has limited translational use due to nephrotoxicity concerns and hastened delivery kinetics [56,57]. Therefore, in the study herein, we utilized a crosslinked CD vector, which operates through a different means than the soluble, monomer form of CD. The polymer exploits the host/guest complexation to temporarily entrap drug in cyclodextrin pockets, reducing the burst release and providing a longer release profile than delivery systems relying on diffusion alone. This “affinity-based delivery” is thought to be an effective approach to increase the delivery of resveratrol both orally and locally [58,59,60]. Notably, CD can be polymerized into various shapes or even onto the surface of a device, thereby providing numerous applications of long-term drug release capabilities in its polymerized form [61,62]. Polymerized cyclodextrin (pCD) can improve drug delivery rates due to its molecular structure and ability to form the aforementioned host/guest inclusion complexes. The results of this study demonstrated that polymerized beta-CD had the highest loading capacity of resveratrol with a steady daily release over 100 days, and the polymerized gamma-CD had the overall highest concentration of resveratrol released at most time points. These initial in vitro findings were encouraging, as they suggested the formulations of beta and gamma polymerized cyclodextrin were optimal for long-term release applications of resveratrol. Nonetheless, gamma polymerized cyclodextrin was chosen for in vivo analysis due to the strong release profiles exhibited. 

Resveratrol has been used as an antioxidant among countless applications such as cancer, ischemia, and neurological diseases and injuries [17,63,64,65,66,67]. Resveratrol has been proposed to have neuroprotective effects against neuroinflammation and oxidative stress by several cellular mechanisms [25]. The suppression of the activation of the NF-κB signaling pathway though either the suppressions of the MAPK signal transduction pathway or the activation of the SIRT1 pathway are among the proposed mechanisms of action [25,68,69,70]. Of interest to the current study is the antioxidative activity of resveratrol through the inhibition of reactive oxygen species production and reactive oxygen species-scavenging properties [71,72]. Accordingly, an antioxidative activity assay was performed using the resveratrol released from the cyclodextrin polymer disks. The assay demonstrated that the resveratrol released from the cyclodextrin was active at scavenging reactive oxygen species for up to 100 days in vitro, although there was a decrease in activity beginning at 60 days. Nevertheless, comparison to the standard curve from free resveratrol indicated that the decrease in antioxidative activity was due to decreased concentrations of resveratrol released from the pCD at the later time points, rather than a decrease in antioxidant activity of the resveratrol. In fact, the concentrations of released resveratrol are within the therapeutic window 5–25 μM (1.14–5.7 μg/mL) to be an effective dosage to modulate oxidative stress and inflammation within neural tissue [10,29,73,74]. Similar antioxidative assays demonstrated these resveratrol levels to be sufficient for antioxidative relief [32]. Furthermore, the continued antioxidant performance from released resveratrol signifies that the loading and release of resveratrol from pCD does not alter the antioxidant properties of resveratrol. Similar work has exhibited a maintenance of antioxidant properties of resveratrol with the complexation with CD [75]. Collectively, these results suggest the promise of loading resveratrol into pCD for the long-term release and effective antioxidative activity. 

Mass spectrometry analysis demonstrated that resveratrol was at the electrode interface following IP delivery and pCD delivery at 18 h post neural probe implantation. A significant decrease in IP delivered resveratrol concentration surrounding the electrode by 7 days in vivo was observed, which supported the known low bioavailability of systemically delivered resveratrol [29,43,76,77]. Remarkably, a steady release of resveratrol from the pCD disks was observed up to seven days in vivo. This agrees with existing in vitro pCD work from the von Recum lab, in which multiple drug types have been delivered at a fairly steady release rate over the course of days/weeks [50,52,78]. Resveratrol metabolites, monosulfate, mono-glucuronide, dihydro monosulfate, dihydro and disulfate, were all evaluated through mass spectrometry as they have been thought to contribute similar physiological benefits as resveratrol [79,80,81]. There was significantly more monosulfate and mono-glucuronide around the implanted probe receiving resveratrol through the pCD disks compared to systemic IP delivery. It has been shown that resveratrol is rapidly metabolized into derivatives containing most abundantly the sulfate and glucuronide forms and found accumulating within tissues [76,77,79,82,83,84,85]. Resveratrol metabolites, particularly sulfates and glucuronides, can perform functions similar to free resveratrol, such as inhibiting COX, COX2 and QR2 enzymes, as well as activating SIRT1 [79,86,87]. Interestingly, hydrolysis of sulfate moieties can result in the contribution of free resveratrol within blood circulation [79,88]. Collectively, this suggests that the pCD is an adequate approach to deliver resveratrol directly to the site of electrode implantation, considering that the resveratrol metabolites can perform numerous functions typically attributed to free resveratrol. 

A unique property of CD polymers is the possibility for refilling the polymer with additional drugs once the initial stock of loaded drug is depleted. Once the drug is introduced to the local area in which the pCD is implanted, the drug will be taken up by the CD polymer based on the same affinity and diffusion characteristics, allowing for refilling of the CD pockets and a second round of drug delivery to occur. It has also been shown that this process is not affected by competition from local biomolecules [78]. This ability for refilling could allow for an indefinite extension of drug release with repeated loading [48,49]. Furthermore, since the pCD loading can be done post polymerization, the CD can be polymerized onto the surface of a device. This will allow for long-term drug release capabilities in its polymerized form from various devices and numerous applications [61,62]. 

## 4. Materials and Methods 

### 4.1. Materials

Lightly epichlorohydrin-crosslinked cyclodextrin prepolymers (alpha-CD, beta-CD, and gamma-CD, 2–15 kDa, average 10 CDs per chain) were purchased from CycloLab Ltd., (Budapest, Hungary). 1,6-diisocyanatohexane (HDI) and 2,2-diphenyl-1-picrylhydrazyl (DPPH) from Sigma Aldrich (St. Louis, MO, USA), N,N-dimethylformamide (DMF, extra dry) from Applied Biosystems (Foster City, CA, USA), and trans-resveratrol (RSV) from PureBulk (Roseburg, OR, USA) were used as received. 

All other reagents, solvents, and chemicals were purchased from Thermo Fisher Scientific (Hampton, NH, USA) in the highest grade available.

### 4.2. Cyclodextrin Modelling for Binding

Molecular structure data files for resveratrol and all CD types were downloaded from the PubChem database. These structures were converted to PDBQT format and loaded into PyRx (Molecular Graphics Laboratory, The Scripps Research Institute, La Jolla, CA, USA) with resveratrol serving as a ligand and CD as a host. The Autodock Vina algorithm was used to predict host/guest affinity interaction energies. Finally, PyMOL (Schrödinger Inc., New York, NY, USA) was used to visualize host/guest models in 3D space [42]. 

### 4.3. Cyclodextrin Fabrication and Loading

#### 4.3.1. Polymerized Cyclodextrin Synthesis

Polymerized CD hydrogel disks were synthesized from lightly crosslinked CD prepolymer [89]. Briefly, 1.5 g of dried CD prepolymer were dissolved in 4.5 mL DMF (33.3% weight/volume). HDI is added in a ratio of 1:0.32 (glucose residue: HDI) and vortexed to combine. Previous work by the von Recum group examined crosslinking densities ranging from 1:0.08 to 1:0.64, and established 1:0.32 to have the desired stiffness and swelling properties, as increasing the crosslink density of CD polymers was found to decrease the water swelling [89]. The solution was then allowed to crosslink for 3 days at room temperature. With two reactive groups on either end, HDI crosslinks to two different CD hydroxyls, allowing cyclodextrin prepolymer to form an insoluble network polymer that functions as a hydrogel. The hydrogel structure remains well hydrated, resulting in good transport properties and consistent drug delivery rates. At the completion of polymerization, the pCD disks (8 or 3 mm diameter) were punched out from a larger polymer network. The characterization of the CD polymers formulated herein were previously performed utilizing thermogravimetric analysis (TGA), differential scanning calorimetry (DSC), tensile testing, and rheological studies [36,89]. For in vitro studies, all three CD types (alpha, beta, and gamma) were used to form CD polymer. For in vivo studies, only gamma-CD polymer was synthesized, as it was found to release the highest amount of resveratrol in in vitro studies. Following disk punching, several washes with DMF, DMF/water and water were completed to remove unreacted CD and HDI. 

#### 4.3.2. Resveratrol Loading into pCD

Resveratrol was dissolved in DMF at 5 wt % and pCD disks were exposed to this loading solution for 3 days at room temperature to allow for drug loading. Optimization of resveratrol concentrations for drug loading into pCD indicated that an increase in resveratrol concentration during drug loading can increase the weight percent loading achieved in pCD polymer disks, therefore determining the loading concentration utilized in the study herein [90]. Loaded pCD was then removed from the solution, blotted, and briefly washed in excess water to remove surface DMF and free (uncomplexed) resveratrol prior to drying at room temperature. Previous work by the von Recum lab demonstrated small molecule interactions with cyclodextrin through FTIR and SPR, hence confirming the pCD to be a drug delivery system [52,91,92].

### 4.4. Resveratrol Release Assay (Quantifying Amount of Resveratrol)

Resveratrol-loaded pCD disks (8 mm) were incubated with 1 mL of phosphate buffer saline (PBS, pH 7.4) at 37 °C with mild shaking. Every 24 ± 4 h, PBS release media was removed for collection, and replaced with fresh PBS to create an infinite sink condition. The collected PBS was analyzed to quantify the concentration of resveratrol at each time point, by spectrophotometric analysis at 321 nm. A standard curve was generated using a known amount of resveratrol in PBS. 

### 4.5. Resveratrol Activity Assay (DPPH)

To estimate the in vitro antioxidative efficacy of resveratrol delivered from pCD, a 2, 2-diphenyl-1-picrylhydrazyl (DPPH) scavenging assay was used. DPPH is a stable free radical which is reduced when scavenged. Briefly, a DPPH (100 μM) in 200 proof ethanol solution was made, following previously reported methods [32,93,94]. Following which, the DPPH solution was incubated with the released resveratrol from the pCD samples in a 1:1 ratio for 30 min at 37 °C in the dark. The absorbance of the solution was measured spectrophotometrically at 516 nm to determine the scavenging activity of resveratrol. A standard curve using free resveratrol concentrations ranging from 0–1000 µg/mL was used to determine % DPPH scavenging. The radical scavenging activity of released resveratrol from pCD disks was calculated using the following equation.

$$\%\;DPPH\;Scavenging\;Activity=(\frac{A-B}{A}*100)$$

Here, (*A*) is the absorbance value of the neat DPPH solution and (*B*) is the absorbance value of the DPPH solution in the presence of the pCD released resveratrol. In order to ensure consistency and repeatability, all samples were tested in triplicate. 

### 4.6. Surgery for IME Implantation

In this study, twenty-seven adult male Sprague Dawley rats (8–10 weeks old, ~225 gm) were used. These animal procedures were approved by the Institutional Animal Care and Use Committee (IACUC) at the Louis Stokes Cleveland Department of Veterans Affairs Medical Center. Animals were implanted with neural probes in the motor cortex for either 1 h, 18 h or 7 days. Animals receiving resveratrol through an intraperitoneal injection were compared to animals receiving resveratrol through local gamma-CD polymer delivery and to control animals that did not receive any resveratrol. There were three animals used per group and at each time point.

For the surgical procedures, rats were anesthetized in an isoflurane chamber (3% in 1.5 L/min O_2_) for about four minutes. Once the surgical plane of anesthesia was reached, anesthesia was maintained with isoflurane at 2.5% by a nose cone. The animal’s head around the surgical site was shaved, and Marcaine (0.25%) was applied subcutaneously around the surgical site. Carprofen (5 mg/kg) and Cefazolin (25 mg/kg) were applied subcutaneously as analgesics and antibiotics, respectively. The animal was placed onto a stereotaxic frame with 1%–2.5% isoflurane flowing through the nosecone to maintain anesthesia. The surgical site was sterilized with alternating cotton tipped applicators of chlorhexidine gluconate (CHG) and isopropanol scrubs. Animal body temperature was maintained on a circulating water pad and vitals (body temperature, heart and respiratory rate, and oxygen levels) were monitored using a MouseSTAT^®^ Pulse Oximeter & Heart Rate Monitor, (Kent Scientific Corp., Torrington, CT, USA). 

On the scalp, an incision was made down the midline and the skin was pulled back to get a clear view of the skull. The periosteum was cleaned off using cotton tipped applicators and gauze. Next, hydrogen peroxide was applied to the skull surface using a cotton tipped applicator to dehydrate the skull. The skull was then primed using Vetbond animal tissue adhesive. With a sterile ruler and a scalpel, the area to be drilled was marked, 2 mm lateral to the midline and 2 mm anterior to the bregma (motor cortex). Using a 2 mm drill bit, the skull was drilled to gain access to the dura. A fine 45° angle dura pick was used to reflect the dura and uncover the brain. A resveratrol loaded cyclodextrin disk was placed into the craniotomy for the animals receiving local delivery of resveratrol. Implants were placed into the cortex (through the cyclodextrin disk for respective animals), approximately 2 mm deep, manually, while avoiding vasculature. Kwik-Cast (World Precision Instruments, Sarasota, FL, USA) was applied over the implant site to keep the brain insulated. Teets cold cure dental cement was used to construct a stable headcap covering the implant hole and tab. The skin was pulled back together and sutured closed with 5-0 monofilament polypropylene suture (Henry Schein, Melville, NY, USA), and coated with triple antibiotic ointment. Analgesia and antibiotics were administered for three days postoperatively. 

### 4.7. Intraperitoneal Delivery of Resveratrol 

Resveratrol (MegaResveratrol, Inc., (Danbury, CT, USA) was weighed in 50 mg aliquots in centrifuge tubes and autoclave sterilized at 121 °C for 20 min. Assays measuring resveratrol quantification and antioxidant activity confirmed no differences between the autoclaved sterile resveratrol and the non-sterile resveratrol (Figure S3). A stock solution of resveratrol was prepared in a sterile cell culture hood by dissolving the autoclaved resveratrol at 50 mg/mL in 50% syringe filtered ethanol (diluted from 95% in sterile water). Based on the animal weight, the resveratrol stock solution was diluted to a concentration of 30 mg/kg resveratrol in a sterile syringe. A final concentration of 2% ethanol was then achieved by withdrawing the appropriate amount of sterile saline solution directly into the syringe. The syringes were prepared immediately before injections and were slightly heated using a hot air drier prior to injection to increase solubility. Adhering to published protocols [10,29], animals received an IP injection of resveratrol 16–24 h prior to surgery as well as directly following the implantation of the neural probe.

### 4.8. Bioavailability Analysis

#### 4.8.1. Sample Preparation 

Animals were euthanized using CO_2_, followed by decapitation and brain harvesting. A 3 mm biopsy punch was utilized to collect tissue samples around the implant site for bioavailability analysis. Biopsied tissue samples were homogenized in 1 mL of ethanol solution using a Tissue-Tearer at 10,000 rpm speed (BioSpec Products Inc, Bartlesville, OK, USA). Following which, homogenates were centrifuged at 2500 rpm for five minutes, and the aqueous supernatant was transferred to clean Eppendorf tubes further analysis.

#### 4.8.2. Liquid Chromatography-Mass Spectrometry (LC-MS)

As a means for quantifying resveratrol in brain tissue, samples were sent out for LC-MS. Supernatant aliquots (200 µL) were shipped on ice to PhenoSwitch Bioscience (Sherbrooke, QC, Canada). Supernatants were dried under nitrogen and then reconstituted with 200 µL of 50% acetonitrile and 50% water containing 10 mM ammonium acetate and 10 ng/mL of internal standard—Resveratrol-13C6. Standard curves were generated by spiking a known amount of resveratrol standard into control tissue samples (animals not receiving resveratrol) and 10 ng/mL of internal standard and were utilized to quantify resveratrol. Known metabolites of resveratrol (mono-glucuronide, monosulfate, disulfate, dihydro monosulfate, and dihyro) were evaluated utilizing LC-MS/MS using reported precursor and product ion. Their identity was validated at the MS/MS level. The absolute amount of metabolite was estimated based on the ratio over resveratrol internal standards signal. 

Acquisition was performed with an ABSciex TripleTOF 5600 (Sciex, Foster City, CA, USA) equipped with an electrospray interface with a 50 μm iD capillary and coupled to an Eksigent μUHPLC (Eksigent, Redwood City, CA, USA). Analyst TF 1.7 software was used to control the instrument and for data processing and acquisition. The source voltage was set to −4.5 kV and maintained at 350 °C, curtain gas was set at 30 psi, gas one at 23 psi and gas two at 30 psi. Acquisition was performed in negative product ion mode. The following precursor ions were used for quantification: 227.07 – 185.05 (resveratrol), 233.08 – 191.07 (internal standard). Separation was performed on a reversed phase Luna Omega Polar C18 column 1 mm i.d., 1.6 μm particles, 50 mm long (Advance Materials Technology, Wilmington, DE, USA) which was maintained at 50 °C. Samples were injected by loop overfilling into a 5 μL loop. For the 2 min LC gradient, the mobile phase consisted of the following solvent A (10 mM NH4 acetate in water) and solvent B (10 mM NH4 acetate in acetonitrile) at a flow rate of 50 μL/min. Quantification was done using the area under the curve with the Sciex MultiQuant software (Framingham, MA, USA).

### 4.9. Statistical Analysis

If not otherwise stated, experiments were carried out in triplicate for statistical analysis (*n* = 3). Data displayed are the mean of each condition, and error bars represent the standard deviation of the triplicate set. ANOVA was done using Prism to determine reported statistical significance. Significance is defined to be a *p*-value < 0.05. 

## 5. Conclusions 

The objective of this pilot study was to present a proof of concept approach to locally deliver resveratrol to the neural probe implantation site, utilizing a novel polymerized cyclodextrin disk loaded with resveratrol. In vitro analysis of the pCD model demonstrated consistent, controlled release of resveratrol from the pCD disks for 100 days, with a slight decrease of release rate around day 60. Evaluation of the antioxidant activity of the released resveratrol discovered stable, free radical scavenging activity for 60 days, which was related to the drop off in released resveratrol around day 60. In vivo analysis of the pCD localized resveratrol delivery system verified a continuous delivery of resveratrol to the microelectrode implantation site for up to 7 days. Thus, the delivery system presented here collectively provides support for the promise of a potential localized, sustained drug delivery system to the brain. Further investigation will include the optimization of reloading pCD disks systemically, circumventing the need for a secondary invasive operation, as well as investigation of several therapeutic drugs delivered in a temporal pattern, deemed necessary to treat the dynamic tissue response to implanted neural microelectrodes. 

## Supplementary Materials

Supplementary materials can be found at https://www.mdpi.com/1422-0067/21/10/3579/s1.

## Abbreviations

CD	Cyclodextrin
pCD	Polymerized cyclodextrin
