# Supplementary material for: Resveratrol Delivery from Implanted Cyclodextrin Polymers Provides Sustained Antioxidant Effect on Implanted Neural Probes
Source: Int J Mol Sci. 2020 May 19;21(10):3579. doi: 10.3390/ijms21103579 (PMC7279014; doi:10.3390/ijms21103579)
Supplement: Supplementary file 1 [file ijms-21-03579-s001.pdf]

Supplemental Information/Materials

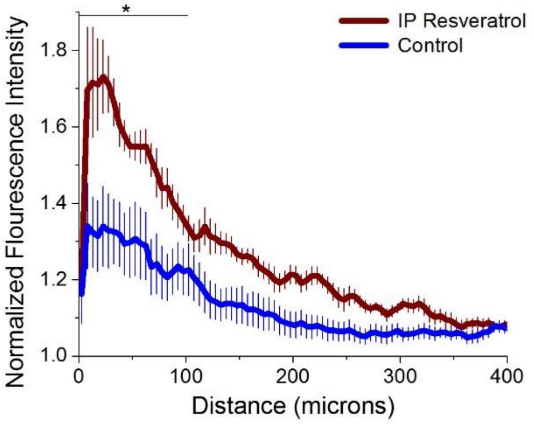

Figure S1. Effect of every other day intraperitoneal resveratrol administration on astrocyte reactivity. There were significantly more reactive astrocytes at two weeks post implantation, within the first 100 microns form the implant site in the animals receiving IP delivery of resveratrol every other day, compared to control animals not receiving any resveratrol.

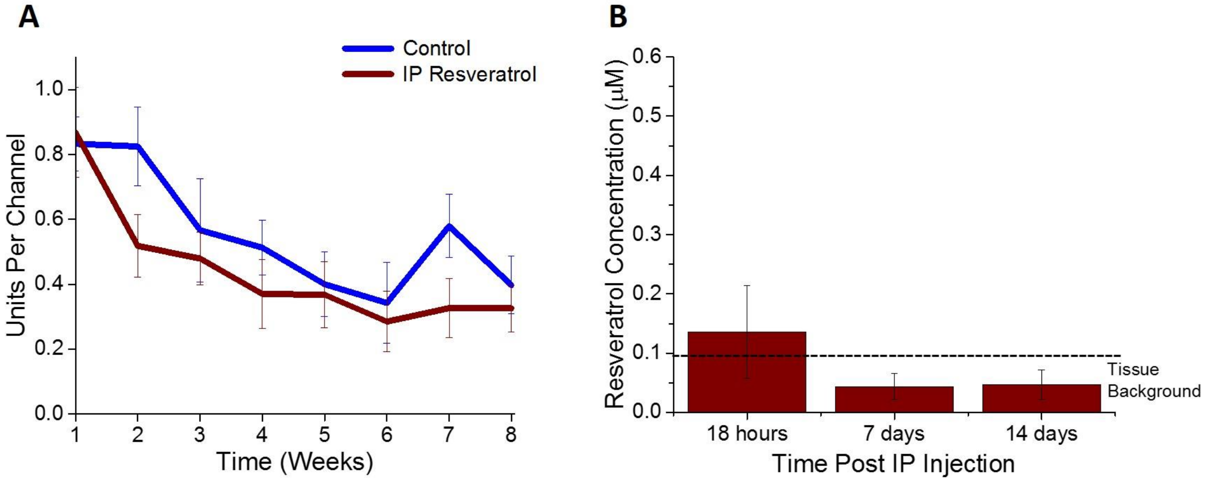

Figure S2. Effect of biweekly intraperitoneal resveratrol administration on electrophysiological recording quality. (**A**) Recording performance of biweekly IP resveratrol administration treated animals (red) compared to control animals (blue). There were no significant differences between the resveratrol group and the control group observed with the number of units recorded per channel. Mass Spec analysis of tissue extracted adjacent the microelectrodes showed low bioavailability of resveratrol (**B**).

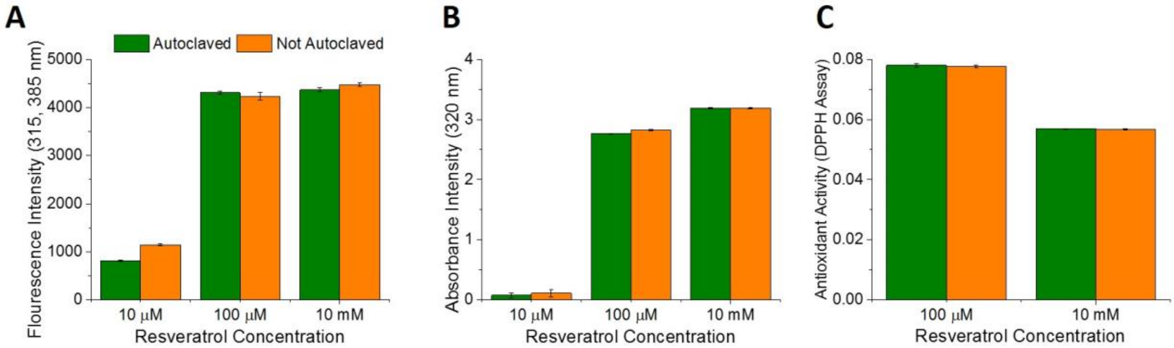

Figure S3. Effect of autoclaving resveratrol on biological activity. Quantitative analysis comparing autoclaved sterile resveratrol (green) to non-sterile resveratrol (orange) was evaluated through (**A**) fluorescence intensity and (**B**) absorbance. Autoclaving resveratrol did not exhibit any differences in the quantification of resveratrol. (**C**) Antioxidant activity was quantified through DPPH assay to observe if autoclaving had any effect on the biological activity of resveratrol. There were no observed differences in antioxidant activity due to sterilization of resveratrol through autoclaving.
